# Editorial: The Cell Types of Fibrosis

**DOI:** 10.3389/fphar.2015.00311

**Published:** 2016-01-22

**Authors:** Lynne A. Murray

**Affiliations:** MedImmune Ltd.Cambridge, UK

**Keywords:** chronic lung disease, tissue fibrosis, progressive decline, autoimmunity, unmet medical need

Fibrosis is a symptom that is present in multiple diseases. It is the common scarring endpoint that is associated with the excessive generation and accumulation of collagen and other matrix proteins. This increase in matrix causes an increase in tissue stiffness, which impacts normal tissue function such as causing impaired gas exchange in the lung, reduced urinary filtration, and output in the kidney, or cirrhosis and portal hypertension in the liver. This ultimately often causes a significant impact on health, as highlighted by the “Living with Fibrosis” article by Sleeman et al. in this Research Topic.

There are numerous mechanisms, cell types and mediators associated with the pathology. The cell types commonly found in areas of active fibrosis are those classically associated with a wound healing response, namely tissue fibroblasts and myofibroblasts (*and shown on the front cover*). However, in normal wound healing, there are a number of “stop signals” that switch off the reparative fibrotic response, allowing for the wound to remodel and excessive scar tissue to be absorbed. In contrast, tissue fibrosis associated with chronic disease tends to continue unabated. This Research Topic is focused on the cell types that have been associated with fibrosis and highlights some of the cutting edge research from groups exploring the initiating factors, propagating signals, and resolving pathways that may elucidate therapeutic approaches to fibrotic diseases.

Fibrosis research is a very challenging area, as many animal models have demonstrated key pathways that when modulated demonstrate efficacy pre-clinically, yet have disappointed in patients. This has caused a challenge in the suitability of preclinical models, in particular *in vivo* models and is justified as fibrotic diseases such as IPF or diabetic nephropathy are thought to arise after years of repeated chronic microtrauma. Yet we are using models of acute injury to assess “key pathways.” For example, idiopathic pulmonary fibrosis (IPF) is a disease of aging, with patients often diagnosed later in life, yet we often assess 6–8 week old mice, days or weeks after inducing injury. We are therefore trying to recapitulate chronic disease in an artificially confined laboratory environment. However, we must still appreciate that these models have added a vast amount to our understanding of chronic and complex disease environments and that we will benefit from current research groups seeking to further improve these models. Using primary cells from patients with fibrosis is another key aspect of pre-clinical fibrosis research. These studies have highlighted important differences between healthy cells and fibrotic cells and are a principal pre-clinical tool.

At the disease level, Gardet et al. review the current understanding of the underlying genetic predispositions that make individuals susceptible to fibrosis. There are common risks factors such as environmental causes or drug treatments and even familial cases of fibrosis; however, we are only now appreciating how certain patients may have a pre-determined susceptibility in sporadic fibrosis. These advances in understanding may help identify approaches to prevent fibrosis. Also, at the disease level, Mancini and Sonis review how multiple cell types are modulated during cancer treatments, resulting in tissue fibrosis. This is often an under-appreciated area in fibrosis research; however, it does have a significant impact on cancer patients, both in limiting cancer treatments, and the ensuing fibrosis causing significant morbidity.

A great deal of fibrosis research is focused on resident fibroblasts and myofibroblasts and their role in driving tissue fibrosis. Habiel and Hogaboam describe the heterogeneous nature of fibroblast in the IPF lung. In the liver, Xu et al. also report on heterogeneity within hepatic myofibroblast populations. Another important resident cell type, the epithelium, is known to be altered in IPF, and Camelo et al. review the current understanding and the role this altered phenotype may play in disease. Mutsaers et al. describe another key resident structural cell, the mesothelium, and how this cell type exhibits altered phenotype and responses in fibrotic setting.

The role of inflammation in disease remains controversial, particularly due to the lack of efficacy of immunosuppressant therapies in most fibrotic indications. However, our ability to profile specific inflammatory cell types is improving and thus may open novel therapeutic approaches. One inflammatory cell that is often found in fibrotic tissue is the mast cell, and Overed-Sayer et al. review the controversy that has surrounded this particular cell type in tissue fibrosis. Another cell type described in fibrosis is the “fibrocyte,” often debated due to the lack of consistency of cell markers and cellular compartments between groups when defining these cells. Reese et al. profile the fibrocyte phenotype in on-going experimental lung fibrosis, indicating that circulating fibrocyte markers alter when the cell type transitions into on-going tissue fibrosis.

A number of novel pathways are reported on in this Research Topic. Agarwal's article reviews the expression and function of a diverse group of proteins, the integrins and cadherins. Traditionally these proteins are thought to regulate cell–cell and cell–matrix adhesion, yet novel functions have more recently been described. Kendall and Feghali-Bostwick also eloquently describe novel pathways that may also be central to pro-fibrotic processes, including how matrix proteins regulate fibroblast and myofibroblast function. Lee et al. present data on caveolin-1 regulating bone marrow derived pro-fibrotic cells in skin fibrosis; Peng et al. report on the role and regulation of the fibroblast growth factor 9 (FGF9 pathway in the experimental fibrotic lung; and Ahsan and Mehal describe the importance of a component of the adenosine pathway in liver fibrosis.

The articles contained in this Research Topic highlight that there is no single cell type that drives tissue fibrosis across the organs. The breadth of the articles also highlight the complex nature of fibrosis and the field needs to pull together, across the different organs affected by fibrosis, and appreciate the similarities as well as the diversity, at the author and reviewer level, as described by Dr. Mehal. There may also not be a single pathway that can be modulated that will impact fibrosis across multiple organs, even though the end stage collagen appears very similar. Fundamentally, our understanding on the heterogeneity of disease needs to improve. Previous research using patient samples has often relied on tissue samples from autopsy, highlighting terminal pathways, which may have deteriorated in the tissue due to imprecise collection techniques. Moreover, samples reported in the literature have often been taken at time of diagnosis (i.e., diagnostic biopsies), which again provides a snap shot of disease. We are only just beginning to understand progression of disease using longitudinal samples. However, as our understanding advances and our appreciation of commonalities across fibrotic diseases improves, we may more rapidly and efficiently be able to address these devastating diseases.

## Author contributions

The author confirms being the sole contributor of this work and approved it for publication.

### Conflict of interest statement

The author declares that the research was conducted in the absence of any commercial or financial relationships that could be construed as a potential conflict of interest.

